# Contribution of the drought tolerance‐related *Stress‐responsive NAC1* transcription factor to resistance of barley to Ramularia leaf spot

**DOI:** 10.1111/mpp.12173

**Published:** 2014-08-25

**Authors:** Graham R. D. McGrann, Andrew Steed, Christopher Burt, Rachel Goddard, Clea Lachaux, Anuradha Bansal, Margaret Corbitt, Kalina Gorniak, Paul Nicholson, James K. M. Brown

**Affiliations:** ^1^ Department of Crop Genetics John Innes Centre Norwich Research Park Norwich NR4 7UH UK; ^2^ Crop Protection Team Crop and Soil Systems Group SRUC West Mains Road Edinburgh EH9 3JG UK; ^3^ Present address: Crop Protection Team Crop and Soil Systems Group SRUC West Mains Road Edinburgh EH9 3JG UK; ^4^ Present address: RAGT Seeds Ltd Grange Road Ickleton Essex CB10 1TA UK

**Keywords:** biotroph, endophyte, hemibiotroph, necrotroph, plant–pathogen interaction, senescence, transgenic resistance

## Abstract

*NAC* proteins are plant transcription factors that are involved in tolerance to abiotic and biotic stresses, as well as in many developmental processes. *Stress‐responsive NAC1* (*SNAC1*) transcription factor is involved in drought tolerance in barley and rice, but has not been shown previously to have a role in disease resistance. Transgenic over‐expression of *HvSNAC1* in barley cv. Golden Promise reduced the severity of Ramularia leaf spot (RLS), caused by the fungus *Ramularia collo*‐*cygni*, but had no effect on disease symptoms caused by *Fusarium culmorum*, *Oculimacula yallundae* (eyespot), *Blumeria graminis* f. sp. *hordei* (powdery mildew) or *Magnaporthe oryzae* (blast). The *HvSNAC1* transcript was weakly induced in the RLS‐susceptible cv. Golden Promise during the latter stages of *R. collo*‐*cygni* symptom development when infected leaves were senescing. Potential mechanisms controlling *HvSNAC1*‐mediated resistance to RLS were investigated. Gene expression analysis revealed no difference in the constitutive levels of antioxidant transcripts in either of the over‐expression lines compared with cv. Golden Promise, nor was any difference in stomatal conductance or sensitivity to reactive oxygen species‐induced cell death observed. Over‐expression of *HvSNAC1* delayed dark‐induced leaf senescence. It is proposed that mechanisms controlled by *HvSNAC1* that are involved in tolerance to abiotic stress and that inhibit senescence also confer resistance to *R. collo*‐*cygni* and suppress RLS symptoms. This provides further evidence for an association between abiotic stress and senescence in barley and the development of RLS.

## Introduction

Barley is the fourth most important cereal crop, grown in diverse environments worldwide (Newton *et al*., 2011). Barley yields are threatened by a wide range of biotic stresses, such as pests and diseases (Walters *et al*., 2012). As agricultural environments evolve through altered agronomic practices and climate change, crops such as barley are predicted to be at risk not only from current biotic stresses, but also from previously unrecognized pest and disease problems (Newton *et al*., 2011; West *et al*., 2012). Unfavourable environmental conditions caused by abiotic stresses, such as drought, flooding, extreme temperature, salinity and nutrient stress, can also cause yield losses that can be in excess of 50% (Bray *et al*., 2000). To maintain food production, crop protection needs to guard against losses caused not only by recognised diseases and abiotic stresses, but also by new and emerging threats.

Ramularia leaf spot (RLS), caused by the ascomycete fungus *Ramularia collo*‐*cygni*, is a newly important disease of barley across Europe (Walters *et al*., 2008). The fungus is seed borne, but can also be spread by airborne spores that germinate on the leaf surface and penetrate through stomata (Havis *et al*., 2014; Stabentheiner *et al*., 2009). *Ramularia collo*‐*cygni* lives as an asymptomatic endophyte during crop development, but can switch lifestyle late in the growing season to become a necrotrophic pathogen causing significant yield losses (Oxley and Havis, 2004; Walters *et al*., 2008). The expression of disease symptoms is associated with an overall decline in the host antioxidant system (Schützendübel *et al*., 2008) and progression of the disease results in an early onset of leaf senescence (Oxley *et al*., 2008). However, the precise conditions that contribute to the fungal transition from endophyte to pathogen are not fully understood. Exposure of barley plants to abiotic stresses, such as high light, results in enhanced RLS symptom development, implying that plant stress may be important to the development of this disease (Makepeace *et al*., 2008; Peraldi *et al*., 2014). If abiotic stress factors are required to elicit the fungal transition from endophyte to pathogen, crops with enhanced tolerance to abiotic stress may also be more resistant to RLS.

Abiotic and biotic stress factors elicit somewhat different plant responses, but some components involved in resistance to different types of stress may be shared (Atkinson *et al*., 2013; Atkinson and Urwin, 2012; Narsai *et al*., 2013). The identification and characterisation of genes that control tolerance to multiple abiotic and biotic stress factors could help to reveal the mechanisms that enable plants to cope with stress, as well as being potential candidates for use in plant breeding programmes. Transcription factors act as molecular switches involved in the regulation of plant developmental programmes and responses to diverse stresses. One of the largest families of transcription factors is the plant‐specific NAC [no apical meristem (NAM), *Arabidopsis thaliana* transcription activation factor (ATAF1/2) and cup‐shaped cotyledon (CUC2)] transcription factor superfamily. NAC transcription factors are involved in the regulation of many developmental processes, including secondary cell wall biosynthesis, senescence and biotic and abiotic stress tolerance (Puranik *et al*., 2012). *NAC* genes can also promote and regulate reactive oxygen species (ROS) metabolism and homeostasis (Lee *et al*., 2012; Wu *et al*., 2012; You *et al*., 2013). Functional diversification within the NAC protein family predates the separation of the monocot and dicot lineages (Nuruzzaman *et al*., 2010) and many different NAC transcription factors have been characterised in cereals with various functions (Distelfeld *et al*., 2012; Uauy *et al*., 2006). Some cereal NAC transcription factors regulate responses to drought, cold and salinity stresses, whereas others act against pathogens (Chen *et al*., 2013; Jensen *et al*., 2007; Nakashima *et al*., 2007; Sun *et al*., 2013; Xia *et al*., 2010). However, many *NAC* genes respond to both abiotic and biotic stresses, and the regulation of multiple stresses appears to be a common function of NAC transcription factors, indicating a degree of functional redundancy within this gene family (Nuruzzaman *et al*., 2010).

*NAC* genes have been used in transgenic approaches to enhance abiotic stress tolerance in cereals. *Stress‐responsive NAC1* (*SNAC1*) was first characterised in transgenic rice (*OsSNAC1*), demonstrating a positive role for this transcription factor in drought and salt tolerance (Hu *et al*., 2006). *OsSNAC1* regulates abiotic and oxidative stress tolerance by enhancing the expression of stress‐responsive genes (Hu *et al*., 2006) and by targeting genes that control stomatal closure and ROS homeostasis (You *et al*., 2013). Over‐expression of the orthologous barley gene *HvSNAC1* also enhanced drought tolerance in barley plants (Al Abdallat *et al*., 2014).

In this article, we demonstrate that SNAC1 has a previously unknown positive function in plant–pathogen interactions. Over‐expression of *HvSNAC1* in barley reduces the severity of RLS symptoms as well as fungal colonisation by *R. collo‐cygni*. This appears to be related to the delayed senescence of *HvSNAC1* over‐expression lines rather than alterations in antioxidant capacity or sensitivity to ROS. The specificity of the response to RLS and the link between *HvSNAC1* over‐expression and enhanced leaf longevity provide further insights into the involvement of senescence in the interaction between barley and RLS.

## Results

### Disease development on *HvSNAC1* over‐expression lines

**Figure 1:**
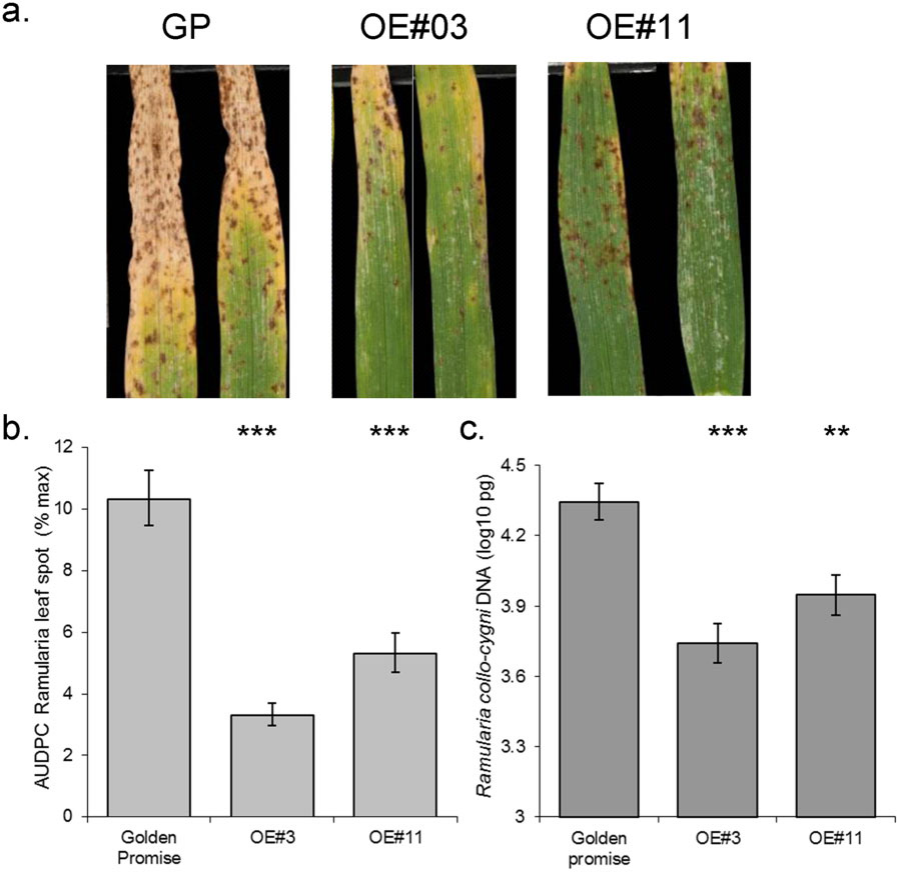
Development of Ramularia leaf spot (RLS) in transgenic *HvSNAC1* over‐expression (OE) barley lines. (a) Disease symptom development at 21 days post‐inoculation (dpi) on Golden Promise (GP), OE#3 and OE#11. (b) Area under the disease progress curve (AUDPC) of RLS. (c) *Ramularia collo*‐*cygni* DNA in leaves of transgenic and wild‐type plants at 21 dpi. Error bars indicate ±1SE. ***P < 0.001 and **P < 0.01 for comparison of means of OE lines with GP.

The role of *HvSNAC1* in defence against fungal diseases was explored using two independent transgenic barley cv. Golden Promise (GP) lines, OE#3 and OE#11, which both exhibited consistently elevated *HvSNAC1* transcript levels in seedling prophyll leaves (Fig. S1, see Supporting Information). Disease symptom expression was assessed on plants inoculated with *R. collo*‐*cygni*, as well as the hemibiotrophic fungal pathogens *Oculimacula yallundae*, *Magnaporthe oryzae* and *Fusarium culmorum*, which differ in the duration of biotrophic growth before entering necrotrophic development, ranging from weeks to days, and the obligate biotroph *Blumeria graminis* f. sp. *hordei*. Typical development of *R. collo*‐*cygni* was observed on cv. GP with the wild‐type *HvSNAC1* gene, a barley variety that shows a moderate degree of susceptibility to isolate Rcc09B4. RLS first became visible from 10 to 12 days post‐inoculation (dpi). The number of spots increased over time with lesions coalescing as the leaf began to senesce (Fig. 1a). The development of *R. collo*‐*cygni* symptoms was reduced in both transgenic lines compared with GP (*P* < 0.001; Fig. 1a,b). Significantly smaller amounts of *R. collo*‐*cygni* DNA were recorded in both transgenic lines compared with GP (60%–75% lower than GP; Fig. 1c). No significant differences were observed in *R. collo*‐*cygni* DNA levels in seeds of GP and the two transgenic lines that were used in the inoculation experiments. (*P* = 0.4; results not shown).

**Figure 2:**
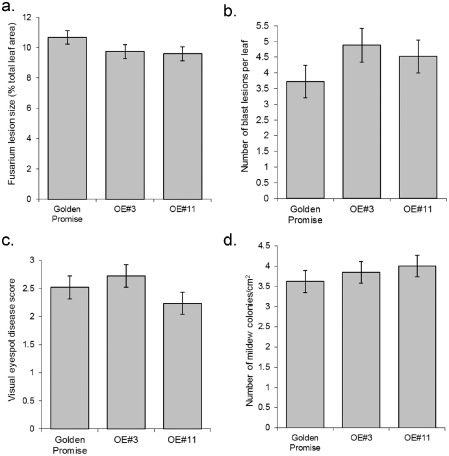
Development of disease symptoms caused by *Fusarium culmorum* (a), *Magnaporthe oryzae* (b), *Oculimacula yallundae* (c) and *Blumeria graminis* f. sp. *hordei* (d) in transgenic *HvSNAC1* over‐expression (OE) barley lines. Error bars indicate ±1SE. None of the mean scores of the OE lines were significantly different from Golden Promise (GP) (*P* > 0.05).

No significant differences were observed in the size of the lesions formed by *F. culmorum* (Fig. 2a; *P* = 0.2) or the number of lesions formed by *M. oryzae* (Fig. 2b; *P* = 0.3) on the leaves of either transgenic line compared with GP. Over‐expression of *HvSNAC1* also had no significant effect on disease symptom development of the stem base eyespot disease caused by *O. yallundae* (Fig. 2c; *P* = 0.3) or on colony formation by *B. graminis* f. sp. *hordei* (Fig. 2d; *P* = 0.8).

### Expression analysis of *HvSNAC1* transcript following *R. collo*‐*cygni* inoculation

**Figure 3:**
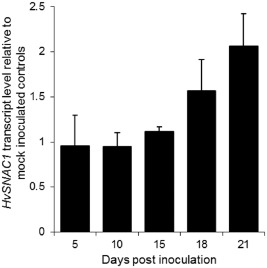
Quantitative reverse transcription‐polymerase chain reaction (qRT‐PCR) time course analysis of *HvSNAC1* transcript expression in wild‐type Golden Promise leaves following infection with *Ramularia collo*‐*cygni*. Transcript levels are presented relative to mock‐inoculated controls. Error bars indicate SE.

Time course analysis of *HvSNAC1* transcript expression during *R. collo*‐*cygni* infection in GP leaves indicated that *HvSNAC1* was not differentially regulated during the early asymptomatic stages of the *R. collo*‐*cygni* infection process, up to 10 dpi (Fig. 3). A small increase in *HvSNAC1* transcript level was observed at the later stages of infection when leaves exhibited severe symptoms and had begun to senesce (Fig. 1a and 3).

### Effect of *HvSNAC1* over‐expression on leaf antioxidant transcript levels and sensitivity to ROS‐induced cell death

**Figure 4:**
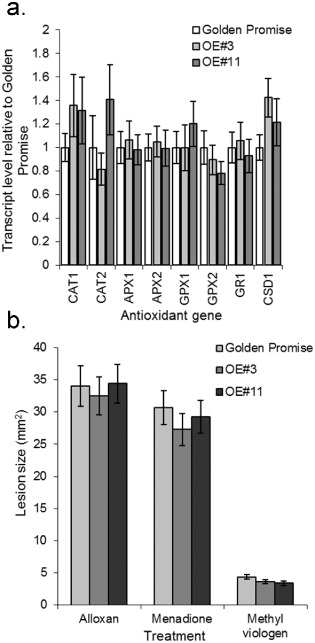
Effect of *HvSNAC1* over‐expression on barley redox system. (a) Quantitative reverse transcription‐polymerase chain reaction (qRT‐PCR) analysis of the constitutive transcript levels of the major reactive oxygen species scavengers in *HvSNAC1* over‐expression lines. Transcript levels are presented relative to wild‐type Golden Promise leaves. APX1, ascorbate peroxidase 1; APX2, ascorbate peroxidase 2; CAT1, catalase 1; CAT2, catalase 2; CSD1, copper/zinc superoxide dismutase 1; GPX1, glutathione peroxidase 1; GPX2, glutathione peroxidase 2; GR1, glutathione reductase 1. Error bars indicate SE (b) Lesion development caused by the reactive oxygen species donors alloxan, menadione and methyl viologen in transgenic *HvSNAC1* over‐expression barley lines. Error bars indicate ±1SE.

The onset of RLS symptoms in the field has been associated with an overall decline in the host antioxidant system (Schützendübel *et al*., 2008). Transcript profiling of the major barley ROS scavengers, including catalases (CAT1 + CAT2), ascorbate peroxidases (APX1 + APX2), glutathione peroxidases (GPX1 + GPX2), glutathione reductase (GR1) and copper/zinc superoxide dismutase (CSD1), was used to assess the effect of *HvSNAC1* over‐expression on the antioxidant system of barley. Constitutive gene expression levels of all of the antioxidant transcripts tested were similar in both transgenic lines to those in GP (Fig. 4a). The role of *HvSNAC1* over‐expression on ROS‐induced cell death was examined by testing the effect of different ROS donors on cell death lesion formation in lines OE#3 and OE#11 relative to GP. No significant differences in lesion size produced by the hydrogen peroxide donor alloxan (*P* = 0.9), the mitochondrial superoxide donor menadione (*P* = 0.7) and the chloroplastic superoxide donor methyl viologen (*P* = 0.1) were observed between either of the transgenic lines and GP (Fig. 4b).

### Effect of *HvSNAC1* over‐expression on leaf senescence and stomatal conductance

**Figure 5:**
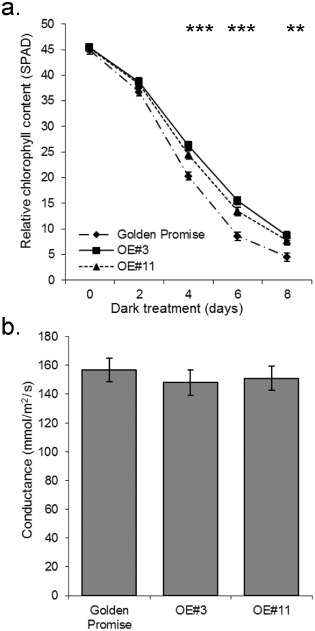
Effect of *HvSNAC1* over‐expression (OE) on dark‐induced senescence (a) and stomatal closure (b) compared with Golden Promise (GP). Error bars indicate ±1SE. ***P < 0.001 and **P < 0.01 for comparison of means of OE lines with GP.

In the field, RLS symptoms become visible during the later developmental stages of the host as the plant begins to flower and the leaves start to senesce (Schützendübel *et al*., 2008). It is not currently known whether leaf senescence is a cause or consequence of disease. Dark‐induced senescence, a model system used to study leaf senescence in plants (Gan and Amasino, 1997), was used to test the effect of over‐expression of *HvSNAC1* on leaf senescence. All three lines showed a decline in relative chlorophyll content over time (Fig. 5a, *P* < 0.001). There was no significant difference between the relative chlorophyll contents of GP and the two transgenic lines at day 0 or after 2 days of dark treatment. From day 4 onwards, there was a significantly slower decline in relative chlorophyll content in both over‐expression lines OE#3 and OE#11 compared with GP (*P* < 0.001). This slower decline in relative chlorophyll content was also observed at day 6 (*P* < 0.001) and day 8 (*P* < 0.01) in both transgenic lines, indicating that over‐expression of *HvSNAC1* delays dark‐induced leaf senescence (Fig. 5a).

Given the role of *HvSNAC1* in drought tolerance and that *R. collo*‐*cygni* infects leaves through the stomata, the effect of the over‐expression of *HvSNAC1* on stomatal closure was examined. No significant differences in stomatal conductance between the transgenic lines OE#3 and OE#11 and GP were observed (Fig. 5b, *P* = 0.7).

## Discussion

As a recently emerging problem of barley, the factors that result in the switching of the endophytic fungus *R. collo*‐*cygni* to a necrotrophic pathogen, resulting in disease, are not fully understood. The development of RLS symptoms typically occurs late in the growing season and appears to be associated with the action of environmental stresses on the host (Makepeace *et al*., 2008; Oxley and Havis, 2004; Peraldi *et al*., 2014; Schützendübel *et al*., 2008). *NAC* transcription factors act as central modulators of stress responses that function as transcriptional activators to regulate the expression of stress‐related genes, including those involved in defence, detoxification and redox homeostasis (Hu *et al*., 2006; Nakashima *et al*., 2007; Sun *et al*., 2013). *SNAC1* transcription factors enhance the drought tolerance of cereals (Al Abdallat *et al*., 2014; Hu *et al*., 2006). Here, we show that over‐expression of the barley *SNAC1* gene confers a small increase in resistance to RLS, but has no effect on the interaction between barley and the fungal pathogens *B*. *graminis* f. sp. *hordei*, *F.* c*ulmorum*, *M. oryzae* and *O. yallundae*. This implies that there is a specific interaction between the endophytic parasite *R. collo‐cygni* and *SNAC1*.

Symbiotic interactions between fungal endophytes and plants are frequently benign or beneficial to the host. However, when host conditions become unfavourable for an endophytic lifestyle, these fungi can become pathogenic (Rodriguez and Redman, 2008). The development of RLS symptoms typically occurs following conditions that are expected to alter host ROS status (Makepeace *et al*., 2008; Peraldi *et al*., 2014; Schützendübel *et al*., 2008). Increased cellular ROS concentrations, if not controlled, can result in cell death, which promotes the development of necrotrophic pathogens (Heller and Tudzynski, 2011). *NAC* genes regulate ROS levels in plants under stressful conditions and the rice orthologue of *HvSNAC1*, *OsSNAC1*, regulates ROS homeostasis through interactions with genes, such as *OsSRO1* (You *et al*., 2013). Over‐expression of *HvSNAC1* did not influence directly the regulation of ROS scavenger transcript expression or sensitivity to ROS‐induced cell death (Fig. 4). This implies that the tolerance of transgenic plants over‐expressing *HvSNAC1* to RLS and restriction of the necrotrophic development of the fungus do not result from elevated resistance to ROS‐related damage.

*Ramularia collo*‐*cygni* is transmitted by infected seed (Havis *et al*., 2014) and spore‐borne foliar infections that occur through stomatal openings (Stabentheiner *et al*., 2009). Seed‐borne inoculum contributes to the initial infection, whereas spore‐borne infections later in the season may play a role in secondary infections and fungal dissemination (Havis *et al*., 2014). The relative contribution of infection via both of these modes of transmission towards final disease levels is not fully understood. Stomata form points of entry for the fungus and, following spore or mycelium inoculation, *R. collo*‐*cygni* grows intercellularly with conidiophores emerging from stomata (Stabentheiner *et al*., 2009; Thirugnanasambandam *et al*., 2011). The rice orthologue of *HvSNAC1*, *OsSNAC1*, can regulate stomatal closure (Hu *et al*., 2006) through its action on *OsSRO1* (You *et al*., 2013), but we found no effect of *HvSNAC1* over‐expression in barley on constitutive levels of stomatal closure (Fig. 5b), corroborating the work of Al Abdallat *et al*. (2014). These data suggest that the reduced susceptibility of the *HvSNAC1* over‐expression lines to *R. collo*‐*cygni* is unlikely to be a result of fewer infection events and reduced fungal penetration resulting from more tightly closed stomata. The effect of *HvSNAC1* over‐expression specifically on seed‐borne RLS epidemiology remains to be determined.

Disease development occurs late in the growing season and has been associated with crop senescence (Oxley *et al*., 2008; Schützendübel *et al*., 2008). Over‐expression of *HvSNAC1* delayed dark‐induced leaf senescence in both transgenic lines (Fig. 5a). *HvSNAC1* transcript levels showed a weak induction in GP during the later stages of RLS symptom development (Fig. 3). However, *HvSNAC1* transcripts are induced in senescing flag leaves (Christiansen *et al*., 2011), and so it is unclear whether the increased expression of this gene is a response to stress caused by fungal colonisation or is a consequence of premature leaf senescence associated with the later stages of disease development (Fig. 1a; Oxley *et al*., 2008; Walters *et al*., 2008). NAC transcription factors can positively or negatively affect senescence (Distelfeld *et al*., 2012; Lee *et al*., 2012; Uauy *et al*., 2006; Wu *et al*., 2012; Zhou *et al*., 2013). *TtNAM‐B1* and *OsNAP*, which are related to the *SNAC* subgroup of cereal *NAC* genes (Nuruzzaman *et al*., 2010), promote leaf senescence in wheat and rice (Uauy *et al*., 2006; Zhou *et al*., 2013), respectively, whereas *HvSNAC1* delays the process in barley (Fig. 5a). The results reported here imply that the over‐expression of a gene which suppresses senescence in barley also suppresses the growth of *R. collo*‐*cygni* and the development of RLS symptoms. NAC transcription factors regulate senescence through different processes, including manipulation of ROS and hormone pathways. Positive regulation of senescence by *OsNAP* appears to be associated with jasmonate biosynthesis and signalling pathways (Zhou *et al*., 2013), but the relationship between *SNAC1* and plant hormones is unknown. Whether the effect of *SNAC1* on leaf senescence operates through previously characterised or novel hormone signalling pathways remains to be revealed.

The longevity of green leaf area has been genetically linked to drought tolerance in cereals (Foulkes *et al*., 2007). *NAC* gene mutants that delay senescence in the model plant *Arabidopsis thaliana* also show enhanced tolerance to abiotic stresses, implying an association between senescence and stress (Lee *et al*., 2012; Wu *et al*., 2012). In the field, there is some evidence that leaf senescence promotes RLS (Schützendübel *et al*., 2008), and barley varieties that are highly susceptible to RLS tend to senesce early as a result of the disease (Oxley *et al*., 2008). Although *HvSNAC1* over‐expression lines, which have reduced susceptibility to RLS (Fig. 1), have a small effect on reducing the rate of leaf senescence (Fig. 5), further experiments are required to elucidate the interactions of the signalling pathways regulated by *HvSNAC1* with *R. collo*‐*cygni*, stress and senescence.

Deciphering the signalling networks controlled by *HvSNAC1* will further our understanding of the host processes that contribute to the expression of RLS. *HvSNAC1* over‐expression affected the development of RLS, but had no significant effect on four other diseases (Fig. 2), despite the fact that the *HvSNAC1* transcript was regulated upwards and downwards by infection with *Fusarium* and *B. graminis*, respectively (Al Abdallat *et al*., 2014). Therefore, it appears that *HvSNAC1* has a specific effect on RLS and is not a major factor contributing to the interaction between barley and other diseases (Fig. 2). The association between symptom development of this late season disease, plant stress and senescence implies that the delayed senescence phenotype of the *HvSNAC1* over‐expression lines is involved in the reduced growth of *R. collo*‐*cygni* and expression of RLS symptoms. As a newly important pathogen, little is known about the host genetic components involved in the interaction with *R. collo‐cygni*. Recent evidence that mutant *mlo* alleles, which confer resistance to the biotrophic powdery mildew fungus, increase susceptibility to RLS (McGrann *et al*., 2014) further implies that senescence is involved in the aetiology of RLS. The wild‐type *MLO* gene responds to both biotic and abiotic stresses (Baker *et al*., 2000) and mutant *mlo* alleles accelerate the rate of leaf senescence once the process has begun (Piffanelli *et al*., 2002). Whether the *mlo*‐enhanced susceptibility to RLS is specifically linked to senescence or to one or more of the other pleiotropic effects caused by the *mlo* mutation requires further experimentation. Interpretation of the link between RLS, senescence and stress responses may provide insights into why RLS has recently emerged as an important disease of barley.

## Experimental Procedures

### Plant material

Two transgenic lines, OE#3 and OE#11, from independent transformation events over‐expressing *HvSNAC1* (*HvNAC003*; Christiansen *et al*., 2011) were used in this study (Al Abdallat *et al*., 2014). Seeds were sown in 8 × 8 × 10‐cm^3^ pots in Levington F2 compost medium (Scotts Professional, Ipswich, UK), and grown under a 16 h/8 h day/night photoperiod at day/night temperatures of 18/12 °C provided by 220 μmol/m^2^/s fluorescent lighting in a controlled environment room (Sanyo Gallenkamp PLC, Loughborough, UK).

### Pathogen inoculations

*Ramularia collo*‐*cygni* isolate Rcc09B4 was used to inoculate barley plants following the protocol of Makepeace *et al*. (2008) with the modifications of Peraldi *et al*. (2014). RLS development was scored as the percentage leaf area covered with disease symptoms three to five times over 21 dpi and the area under the disease progress curve (AUDPC) was calculated. Three independent replicate experiments, each containing an individual 8 × 8 × 10‐cm^3^ pot of 10 seeds of each line, were inoculated with *R. collo*‐*cygni*. *Oculimacula yallundae* isolate P149 was inoculated at the stem base of 21‐day‐old plants using the method of Chapman *et al*. (2008). A single experiment was inoculated in a randomised block design, consisting of five blocks, with each block containing five plants of each line. The penetration of stem bases by *O. yallundae* was assessed 8 weeks post‐inoculation using the scale of Scott (1971). *Magnaporthe oryzae* isolate BR32 was inoculated at a concentration of 10^5^ spores/mL in two independent experiments, as described previously (Tufan *et al*., 2009). In each experiment, 10 seeds of each line were sown in individual 8 × 8 × 10‐cm^3^ pots and disease was assessed at 6 dpi as the number of blast lesions present on the second seedling leaf of each plant. The development of *F. culmorum* isolate Fu42 was assessed on detached leaves inoculated with two 5‐μL droplets of 10^6^ conidia/mL amended with 75 μm deoxynivalenol (Chen *et al*., 2009). Photographs of disease lesion development were taken 48 h post‐inoculation and the lesion area was measured using ImageJ software (Abramoff *et al*., 2004). Two independent replicate experiments, each containing a minimum of six replicate leaves of each line, were inoculated. *Blumeria graminis* f. sp. *hordei* isolate CC148 was used to inoculate leaf segments from 14‐day‐old prophyll leaves following the method of Boyd *et al*. (1994). Disease development was assessed at 14 dpi as the number of colonies observed per square centimetre of leaf area in four independent replicate experiments, each consisting of a minimum of eight replicate leaves of each line.

The response of *HvSNAC1* over‐expression lines to each pathogen was analysed separately using a general linear model (GLM) in GenStat v.15 (Payne *et al*., 2009). Each GLM assessed the variation attributable to experimental replicate, block and line, and calculated the predicted mean disease scores on each line. Predicted means were used to compare the disease development of each pathogen on the two transgenic lines and GP with *t*‐tests using the residual standard error of the respective model.

### Quantitative reverse transcription‐polymerase chain reaction (qRT‐PCR) gene expression assays

Constitutive levels of *HvSNAC1* and ROS scavenger gene transcripts were assessed in each of the two transgenic lines compared with GP using qRT‐PCR. RNA was extracted from 14‐day‐old prophyll leaves, processed and converted to cDNA (Colebrook *et al*., 2012). Transcripts were amplified with gene‐specific primers (Table S1, see Supporting Information; Shagimardanova *et al*., 2010) and the Sybr Green JumpStart™ Taq Ready mix system (Sigma‐Aldrich, St. Louis, MO, USA), as described previously (Colebrook *et al*., 2012). cDNA samples were normalised with geNorm (Vandesompele *et al*., 2002) using five reference genes (elongation factor 1α, glyceraldehyde 3‐phosphate dehydrogenase, cyclophilin, ubiquitin, α‐tubulin; Burton *et al*., 2004; McGrann *et al*., 2009; Rostoks *et al*., 2003). Data were collected from three independent replicate experiments, each consisting of at least two independent samples of each line.

Expression of the *HvSNAC1* transcript during the host response to *R. collo*‐*cygni* infection was assessed in GP. Seedlings were inoculated as described above with either Rcc09B4 or potato dextrose broth containing no fungus as the control. Two samples, each consisting of two pooled prophyll leaves from Rcc09B4 and control inoculated plants, were collected at 5, 10, 15, 18 and 21 dpi. Samples were processed as above and the expression of the *HvSNAC1* transcript was compared between Rcc09B4 and control inoculated samples at each time point. Data were obtained from three independent replicate experiments.

### qPCR quantification of *R. collo*‐*cygni* DNA levels *in planta*

In order to estimate the amount of *R. collo*‐*cygni* within host leaves, fungal DNA levels were measured by qPCR (Taylor *et al*., 2010). DNA was extracted from five leaf samples of each line collected at 21 dpi using the DNeasy Plant Mini Kit (Qiagen, Hilden, Germany). Fungal DNA levels in three independent replicate experiments were quantified by qPCR using a CFX96 thermocycler (Bio‐Rad, Hercules, CA, USA). Differences in log_10_ transformed fungal DNA levels between the two transgenic lines and GP were analysed using a GLM in GenStat v.15 with experimental replicate and line as factors. *Ramularia collo*‐*cygni* DNA levels in seeds were assessed using the methods of Havis *et al*. (2014).

### ROS‐induced cell death assays

ROS‐induced cell death assays were performed on barley leaves following the method of Saville (2011). Prophyll leaves from 14‐day‐old plants of each transgenic line and GP were detached and suspended across agar (1% agar w/v supplemented with 100 mg/L benzimidazole; Sigma‐Aldrich) bridges in clear plastic boxes. The sensitivity of each line to ROS‐induced cell death was tested by adding 2 μL of solution, supplemented with 0.5% v/v Tween20, of the following ROS donors to the centre of each leaf: 200 mm alloxan (Sigma‐Aldrich), 100 mm menadione (Sigma‐Aldrich) and 25 μm methyl viologen (Sigma‐Aldrich). Inoculated leaves were stored under constant light (15–20 μmol/m^2^/s) for 96 h at room temperature. After incubation, each box was photographed and the lesion size was measured using ImageJ. ROS‐induced lesion formation was measured from three independent replicate experiments, each consisting of a minimum of 24 replicate leaves of each line for each ROS donor. General linear modelling was used to estimate the effects of replicate experiment and line. Each ROS donor was analysed separately.

### Stomatal conductance measurements

The effect of over‐expression of *HvSNAC1* on stomatal conductance was measured using an AP4 cycling porometer (Delta‐T Devices Ltd, Cambridge, UK) based on the methods described by Prats *et al*. (2006). Leaf water conductance measurements were taken from the prophyll leaf of 5–10 14‐day‐old seedlings of each line. The experiment was performed four times. Data were analysed using a GLM in GenStat v.15 with experiment and line as factors.

### Dark‐induced senescence assay

Dark‐induced senescence in barley leaves was measured following the method outlined by Peraldi (2012). Prophyll leaves from 14‐day‐old seedlings were excised and an indirect measurement of leaf chlorophyll content was taken using a SPAD 502 Plus Chlorophyll Meter (Konica Minolta, Warrington, UK). Three measurements were taken across each leaf to cover the distal, middle and basal portions, and averaged. After the first measurement (day 0), leaves were placed on damp tissue paper in 10‐cm^2^ plastic Petri dishes, covered with aluminium foil and kept in the dark at room temperature to induce senescence. Further measurements were taken on each leaf after 2, 4, 6 and 8 days of dark treatment. Three replicate experiments were performed with measurements taken from six leaves of each line in each experiment. Linear mixed modelling of repeated measurements was used to evaluate differences in SPAD readings between each line at the different time points employing the uniform correlation/split plot in time covariance matrix. Fixed factors included day, experiment, line and the interactions between day and line, and experiment and day. The random factor was the leaf‐by‐day interaction term. Significant differences between lines at different days were subsequently assessed using a *t*‐test.

## Supporting information


**Fig. S1** Quantitative reverse transcription‐polymerase chain reaction (qRT‐PCR) confirmation of constitutive increase in *HvSNAC1* transcript levels in transgenic barley over‐expression (OE) lines. Error bars indicate ± 1SE. ***P < 0.001 and **P < 0.01 for comparison of means of OE lines with Golden Promise (GP).
**Table S1** Quantitative reverse transcription‐polymerase chain reaction (qRT‐PCR) primers used in this study.
