# Supplementary figures and images for: Contribution of the drought tolerance‐related Stress‐responsive NAC1 transcription factor to resistance of barley to Ramularia leaf spot
Source: Mol Plant Pathol. 2014 Aug 25;16(2):201–9. doi: 10.1111/mpp.12173 (PMC4344812; doi:10.1111/mpp.12173)

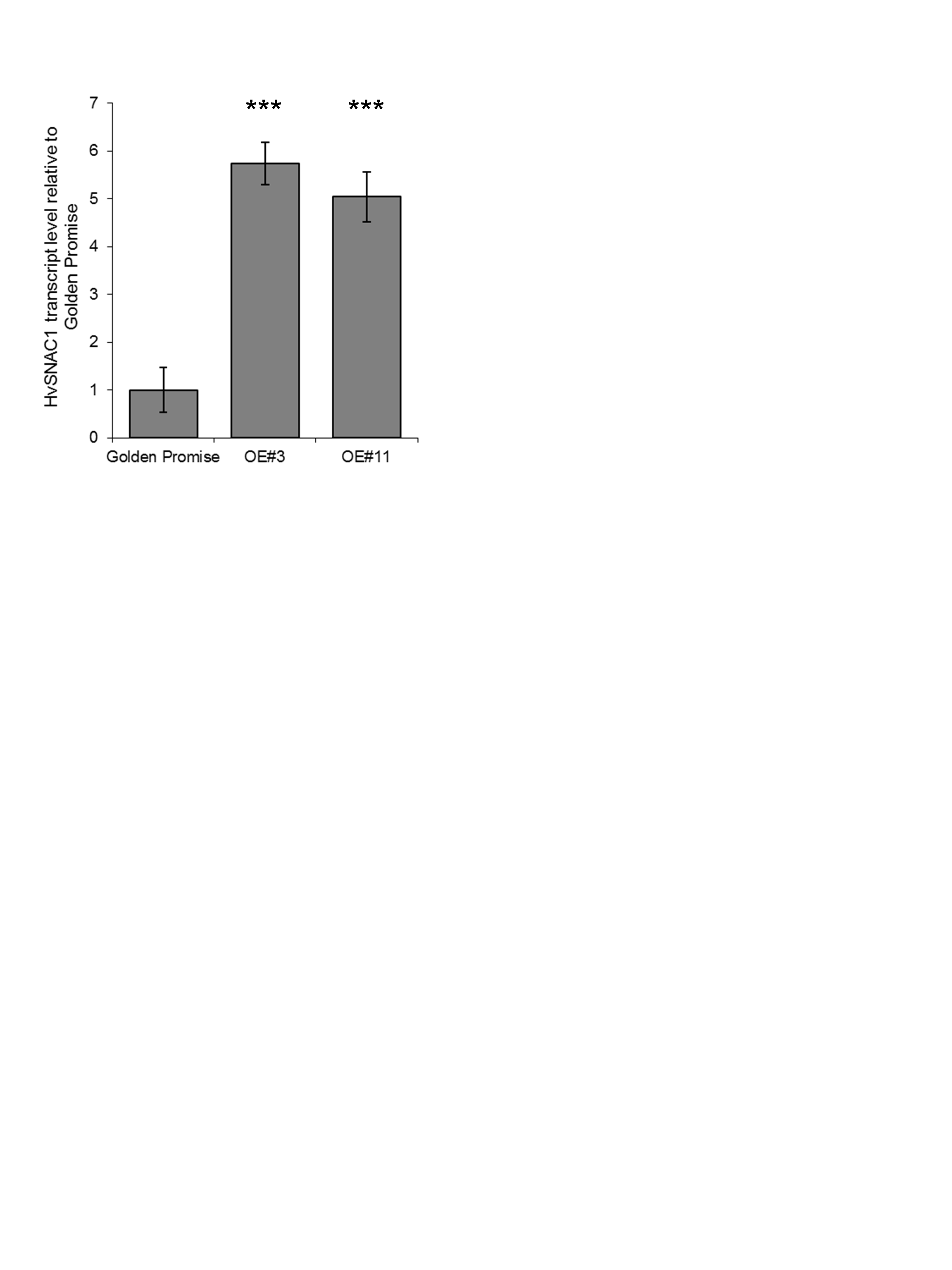

Supplement: Supplementary file 1 — Fig. S1 Quantitative reverse transcription‐polymerase chain reaction (qRT‐PCR) confirmation of constitutive increase in HvSNAC1 transcript levels in transgenic barley over‐expression (OE) lines. Error bars indicate ± 1SE. ***P < 0.001 and **P < 0.01 for comparison of means of OE lines with Golden Promise (GP). Table S1 Quantitative reverse transcription‐polymerase chain reaction (qRT‐PCR) primers used in this study. [file MPP-16-201-s001.zip › mpp_12173-supp-0001-Fig. S1.tif]
