# Supplementary material for: Contribution of the drought tolerance‐related Stress‐responsive NAC1 transcription factor to resistance of barley to Ramularia leaf spot
Source: Mol Plant Pathol. 2014 Aug 25;16(2):201–9. doi: 10.1111/mpp.12173 (PMC4344812; doi:10.1111/mpp.12173)
Supplement: Supplementary file 1 — Fig. S1 Quantitative reverse transcription‐polymerase chain reaction (qRT‐PCR) confirmation of constitutive increase in HvSNAC1 transcript levels in transgenic barley over‐expression (OE) lines. Error bars indicate ± 1SE. ***P < 0.001 and **P < 0.01 for comparison of means of OE lines with Golden Promise (GP). Table S1 Quantitative reverse transcription‐polymerase chain reaction (qRT‐PCR) primers used in this study. [file MPP-16-201-s001.zip › mpp_12173-supp-0001-Table S1.doc]

Table S1 qRT-PCR primers used in this study

| Target | Forward primer | Reverse primer | Amplicon size (bp) |
| --- | --- | --- | --- |
| *HvSNAC1* | AGATGATGGCCACGCTGAT | GAGGCCGTTGTACATGCTCT | 110 |
| *HvCAT1* | CCCGTCTGGAACAACAAC | CCCCGTGCATGAACAAC | 134 |
| *HvCAT2* | CGACGACAAGATGCTGCAGT | TGGTTGTTCTTGAAGCCGC | 122 ^a^ |
| *HvGPX1* | AACGGCAACAATGTTTCTCC | ACAACGTGACCCTCCTTGTC | 119 |
| *HvGPX2* | ACGTGAATGGCAACAATGCT | ATGACATGCCCCTCTTTGTC | 124 |
| *HvGR1* | GGGGCTATAGTGGTCGATGA | AATGCTCCACCTTCCATCAG | 116 |
| *HvAPX1* | CGGAGCTTTTGAGTGGTGACA | CCGCAGCATATTTCTCCACAA | 107 ^a^ |
| *HvAPX2* | CGCCGAGAAGAACTGC | GCCGGTCTTGGTGGC | 82 |
| *HvCSD1* | ACCTCGGAAATGTGACAGC | ACCCTTGCCAAGATCATCAG | 140 |
|  |  |  |  |
| Reference genes | |  |  |
| *HvCyclophilin* | TTGAGGACGAGATAAGGCCAG | GCGACTGACAAGGTGCAAGAG | 120 ^b^ |
| *HvEF1a* | ATGATTCCCACCAAGCCCAT | ACACCAACAGCCACAGTTTGC | 101 ^b^ |
| *HvGAPDH* | CCTTCCGTGTTCCCACTGTTG | ATGCCCTTGAGGTTTCCCTC | 124 ^b^ |
| *HvTUBA* | AGTGTCCTGTCCACCCACTC | AGCATGAAGTGGATCCTTGG | 248 ^c^ |
| *HvUbiquitin* | GCCGCACCCTCGCCGACTAC | CGGCGTTGGGGCACTCCTTC | 219 ^d^ |
|  |  |  |  |

^a^ Shagimardova et al. (2010), ^b^ McGrann et al. (2009), ^c^ Burton et al. (2004), ^d^ Rostoks et al. (2003)
